# Conservation Awareness as a Key Mediator Linking Affective Factors to Species Knowledge in Pre-Service Biology Teachers 

**DOI:** 10.12688/f1000research.188371.1

**Published:** 2026-09-07

**Authors:** Tilal Afian, Ibrohim Ibrohim, Fatchur Rohman

**Affiliations:** 1 Biology Education, Universitas Negeri Malang, Malang, East Java, Indonesia; 2 Biology Education, Universitas Cordova, West Sumbawa Regency, West Nusa Tenggara, Indonesia

**Keywords:** Affective factors, Biology teacher education, Conservation awareness, Conservation education, Environmental education, Mediation analysis, Pre-service biology teachers, Species knowledge.

## Abstract

**Background:**

Species literacy is an important achievement in biodiversity education. The study analyzed the roles of affective dimension and conservation awareness in shaping species knowledge among prospective biology teacher students, using *Lichmera indistincta limbata* as a local species.

**Methods:**

This study employs a quantitative research approach using a survey design to examine the relationships among affective dimensions, conservation awareness, and species knowledge. Partial Least Squares–Structural Equation Modeling (PLS-SEM) is used to test conceptual models through the mediating role of conservation awareness. The research participants are prospective biology teachers registered in the S1 biology education study program in the provinces of West Nusa Tenggara and East Nusa Tenggara, Indonesia. A total of 137 respondents from eight public and private universities participated in this study. All items were measured using a five-point Likert scale. Data collection was done online through Google Forms. The affective domain consists of three constructs, namely attention and care, closeness and appreciation, and a sense of responsibility. The construct of species knowledge was developed based on a species literacy framework that focuses on understanding biological characteristics, ecological roles, and the relevance of species conservation in local contexts.

**Results:**

The results show that attention and concern have a significant effect on conservation awareness, which in turn contributes to species knowledge. Closeness and appreciation have a direct effect on species knowledge, while a sense of responsibility does not show a significant influence. In addition, conservation awareness has been shown to play a significant role as a mediator between concern and species knowledge.

**Conclusions:**

This study highlights that conservation awareness is a key mechanism bridging the affective and cognitive dimensions of species literacy and is important to integrate into the education of prospective biology teachers in the context of local species.

## Introduction

The global biodiversity crisis has placed conservation education as the foundation for environmental sustainability. When ecosystems lose biodiversity, environmental changes such as climate change and pollution occur, making it difficult to restore them to their original state. ^1^ Ecosystem degradation not only disrupts the ecological balance but also threatens human well-being. This problem can reduce genetic diversity and inhibit species’ ability to adapt to various conditions, thereby increasing the risk of local species extinction. ^2^ Biodiversity literacy in this context, especially species literacy, has become the most important component to be developed through biology education in the formal environment. ^3^ Species literacy allows individuals to understand the biological characteristics, ecological roles, and conservation urgency of organisms in their ecosystems.

The results of an empirical study report that elementary school students in the Netherlands can correctly identify only about 35% of native species, indicating a low level of species literacy. ^4^ Similarly, in northern Spain, primary school students are more interested in exotic species than local species. ^5^ This difference provides a gap between species consciousness and knowledge. Prospective biology teachers occupy a strategic position in answering these challenges. They are responsible for educating students in species identification, explaining the role of ecosystems and conservation. ^6^ Activities such as feeding and monitoring birds directly increase their awareness and knowledge of species. ^7^ Activities such as feeding and monitoring birds directly increase their awareness and knowledge of species. ^7^ Students can understand ecological roles and conservation efforts. ^8^

Previous studies have shown that species literacy is influenced by affective and cognitive dimensions. ^9^ Affective factors such as care and concern, closeness and appreciation, and feelings of responsibility are often considered determinants of ecological concern. These relationships include cognitive, emotional, ethical, and spiritual dimensions. ^10^ However, empirical findings on its direct contribution to species knowledge remain inconsistent. ^11^ ^,^ ^12^ Due to the complex levels of ecological understanding, emotional connection with nature, and moral drive to maintain ecosystem survival. ^13^ This suggests the need for a more in-depth explanation of the mechanisms linking affective involvement and species knowledge.

In an empirical study of the influence of conservation awareness on local species in Nepal, most of the population is unaware of the conservation status *of the Lesser Adjutant Crane*, despite knowing its ecological role. ^14^ Most research focuses on environmental issues. Land conversion and forest degradation in Indonesia threaten endemic bird species and complicate conservation efforts. ^15^ Hunting practices increasingly threaten local bird populations, which has an impact on conservation efforts. ^16^ These limitations limit the understanding of how caring for biodiversity is defined by conservation-oriented cognition.

Conservation awareness is theoretically positioned as an affective-cognitive construct that connects attitudes and knowledge. Empirical studies examining conservation awareness as a mediating mechanism between affective factors and species knowledge remain limited among prospective biology teachers. Previous research has highlighted the importance of mindfulness in ecological learning but rarely examined its mediating role using structural models ^17^Another limitation is the lack of research related to endemic species. These limitations make it difficult to research the mediating role of conservation awareness effectively, ^18^ especially by prospective biology teachers. ^19^

*Lichmera indistincta limbata is an endemic bird species*, ^20^ with ecological functions that remain underexplored in species literacy research in universities. ^21^ This gap highlights the need for empirical studies that integrate local biodiversity into species literacy research. The study aims to integrate models by examining affective dimensions, conservation awareness, and species knowledge within a single framework using *Partial Least Squares–Structural Equation Modeling* (PLS-SEM), and to test the mediating role of conservation awareness in shaping the species literacy of prospective biology teacher students through the species *Lichmera indistincta limbata.* The study wanted to find out whether the affective dimension (attention and care, closeness and appreciation, and sense of responsibility) affected conservation awareness in prospective biology students, whether the affective dimension and conservation awareness affected species knowledge, and whether conservation awareness mediated the relationship between affective dimensions and species knowledge.

## Methods

### Research design

This study employs a quantitative research approach using a survey design to examine the relationships among affective dimensions, conservation awareness, and species knowledge. The *Partial Least Squares–Structural Equation Modeling* (PLS-SEM) approach was used to test the proposed conceptual model through the mediating role of conservation awareness. PLS-SEM was chosen because of its suitability for predictive analysis, complex structural models, and relatively small sample sizes without the assumption of strict normality. ^22^

### Participants research

The participants in this study are prospective biology teachers registered in the S1 biology education program in the provinces of West Nusa Tenggara and East Nusa Tenggara, Indonesia. A total of 137 respondents from eight public and private universities participated in this study. The composition of respondents was dominated by women, namely 120 students and 17 male students. This gender distribution reflects the general characteristics of biology education programs in the region. The selection of respondents is due to their strategic role as future biology teachers who will influence students’ understanding and attitudes towards biodiversity and conservation.

### Data collection

Data were collected using a structured questionnaire designed to measure two main domains, namely affective domain and conservation awareness, and species knowledge as outcome variables. All items were measured on a Likert scale, and data were collected online via Google Forms. All items were measured using a five-point Likert scale. Participation was voluntary, and respondents were provided with information about the research objectives and the confidentiality of the data before filling out the questionnaire.

The affective domain consists of three constructs, namely attention and care, closeness and appreciation, and a sense of responsibility. Each construct was measured using several indicators adapted from previous research on species literacy, biodiversity education, and conservation awareness. ^9^ ^,^ ^17^ ^,^ ^23^ The species knowledge construct was developed based on a species literacy framework that focuses on understanding biological characteristics, ecological roles, and the relevance of species conservation in local contexts. ^9^

The results of the reliability test showed high internal consistency, with Cronbach’s Alpha values of 0.958 for attention and concern, 0.940 for closeness and appreciation, and 0.894 for sense of responsibility. The domain of conservation awareness is measured using five indicators that reflect an individual’s understanding and awareness of biodiversity conservation issues. This construct shows excellent reliability, with a Cronbach’s alpha of 0.950. The entire construct meets the criteria for convergent validity and composite reliability, as indicated by the Average Variance Extracted (AVE) > 0.50 and Composite Reliability values >0.70.

### Data analysis

Data analysis was carried out using SmartPLS version 4.1.1.6. The analysis follows two main stages: evaluation of the measurement model and evaluation of the structural model. Convergent validity was assessed using *outer loadings* and *Average Variance Extracted* (AVE), with threshold values of ≥ 0.70 and ≥ 0.50, respectively, ^24^ Discriminant validity was assessed using *cross-loadings* to ensure that each indicator has the highest *loading* on its intended construct. The reliability of the construct was evaluated using *Cronbach’s Alpha* and *Composite Reliability*, with values above 0.70 indicating an acceptable reliability level. ^25^

The structural model was evaluated by examining the path coefficients, the determination coefficient (R ^2^), and the significance of the direct and indirect effects. Significance testing was performed using *the bootstrapping* procedure *.* ^26^ A relationship is considered statistically significant when the t-value exceeds 1.96, and the p-value is below 0.05. ^27^ This threshold is usually used to determine whether the observed effect is unlikely to occur by chance.

## Results

Before structural relationship testing, the measurement model is evaluated to ensure the construct’s validity and reliability. This stage is important for confirming that the latent construct has been measured precisely before proceeding to hypothesis testing using structural models.

### Evaluation of the measurement model

Convergent validity was assessed using outer loading and Average Variance Extracted (AVE). Reliability was evaluated using Cronbach’s Alpha and Composite Reliability (CR). To improve the model’s quality, indicators with outer loadings below 0.70 were removed during the initial estimation stage. The final measurement model only retains indicators that meet the recommended criteria. The Measurement Model Assessment Criteria are presented in Table 1.

**Table 1.** Measurement model assessment.

Construct	Outer Loadings Range	AVE	Cronbach’s Alpha	Composite Reliability
Attention and Concern	0.857–0.913	0.800	0.958	0.959
Closeness and Appreciation	0.936–0.956	0.893	0.940	0.941
Feeling of Responsibility	0.902–0.914	0.825	0.894	0.894
Conservation Awareness	0.837–0.939	0.834	0.950	0.953
Species Knowledge	0.926–0.950	0.878	0.930	0.932

All constructs show satisfactory convergent validity, with AVE values exceeding the recommended threshold of 0.50. This value confirms that the constructs have adequate convergent validity. ^28^ In general, *outer loading* is considered strong if the value is above 0.70. A *high outer loading* value indicates that the indicator is a reliable measure for latent constructs. ^29^ The reliability rating shows strong internal consistency, as Cronbach’s Alpha and *Composite Reliability* values are well above the recommended threshold of 0.70. The reliability rating shows strong internal consistency, as Cronbach’s Alpha and *Composite Reliability* values are well above the recommended threshold of 0.70. Cronbach’s Alpha describes the extent to which the items in an instrument measure the same construct and reflect the degree of correlation between the items, thus providing a more accurate estimate of reliability. ^30^ ^,^ ^31^

These findings show that the measurement instruments used to assess affective dimensions, conservation awareness, and species knowledge in prospective biology teacher students are statistically robust. These results are in line with previous research in biodiversity and environmental education that emphasizes the importance of reliable affective-cognitive measures for assessing species literacy and conservation-related constructs. ^4^ ^,^ ^17^

### Model fit assessment

The model fit was evaluated using several fit indices from SmartPLS version 4.1.1.6, including standardized root mean square residual (SRMR), d_ULS, d_G, chi-square, normed fit index (NFI), and goodness-of-fit (GoF). The results of the model fit assessment are presented in Table 2.

**Table 2.** Model fit assessment.

Parameter	Rule of Thumb	Value	Interpretation
SRMR	< 0.10	0.042	Fit
d_ULS	> 0.05	0.401	Fit
d_G	> 0.05	1.054	Fit
Chi-square	χ ^2^ statistic ≥ χ ^2^ table	560.052 ≥ 31.410	Fit
NFI	Approaching 1	0.827	Fit
GoF	0.10 (small), 0.25 (medium), 0.36 (large)	0.854	Fit

### Structural model fit and predictive accuracy

The overall quality of the structural model was evaluated using multiple fit indices generated by SmartPLS 4.1.1.6. The model shows a good fit to the data, as indicated by an SRMR of 0.042, well below the recommended threshold of 0.10. This value indicates excellent model fit, confirming that the hypothesized relationship in SEM is consistent with the observed data. ^32^ Additional conformity indices, including d_ULS and d_G, also met the recommended criteria, thus indicating an acceptable difference between the empirical correlation matrix and the correlation matrix implied by the model. This combined approach allows for a multidimensional evaluation of the model’s adequacy, providing a comprehensive picture of how well the model is compliant across various conformity metrics. ^33^ Careful evaluation of this index underscores the influential application of SEM in empirical research.

The NFI value of 0.827 is close to the ideal value of 1. This value suggests that the modeled relationships are relevant and correlated well with the data, reflecting strong support for relationships between constructs. ^34^ ^,^ ^35^ A GoF value of 0.854 indicates strong overall model performance. A GoF value of 0.854 indicates strong overall model performance. GoF measures how well the model’s predictive performance aligns with the observed data. ^36^ These results confirm that such structural models are feasible to use for hypothesis testing. Similar model suitability thresholds and interpretations have been consistent with the application of PLS-SEM in educational and environmental research. ^27^

### Coefficient of determination (R ^2^)

The predictive accuracy of the structural model is assessed using the coefficient of determination (R ^2^) for the dependent variable construct, as presented in Table 3. The results showed that the model explained 89.4% of the variance in conservation awareness and 83.7% of the variance in species knowledge. An R ^2^ value above 0.8 indicates that more than 80% of the variation in the dependent variable can be explained by Model. ^37^ These values indicate strong predictive accuracy, suggesting that the affective constructs included in the model provide substantial explanations for conservation awareness and species knowledge. High R ^2^ values in biodiversity education research indicate that affective and awareness-related variables play an important role in shaping ecological understanding. ^38^ For prospective biology teachers, these findings show that structured affective-cognitive processes strongly influence species literacy.

**Table 3.** Coefficient of determination (R ^2^).

Variable Dependen	R ^2^	Adjusted R ^2^
Conservation Awareness	0.894	0.892
Species Knowledge	0.837	0.833

### Path coefficients and hypothesis testing (Direct Effects)

The significance of the hypothesized direct relationship was examined using the bootstrapping procedure, as shown in Table 4. Based on the results of the structural model test through the bootstrapping procedure in Table 4, there are several significant and insignificant relationships between constructs. Attention and concern had a positive and significant effect on conservation awareness (β = 0.767; t = 7.399; p < 0.001), indicating that the higher an individual’s concern for biodiversity, the higher their conservation awareness. In contrast, attention and concern had no significant effect on species knowledge (β = 0.091; p = 0.270), so the hypothesis linking the two variables was not supported. This shows that affective involvement does not directly increase knowledge without going through intermediate variables.

**Table 4.** Path coefficient bootstrapping direct effect results.

Path	β	t-value	p-value	Result
Attention and Concern →Conservation Awareness	0.767	7.399	0.000	Supported
Attention and Concern → Species Knowledge	0.091	0.613	0.270	Not supported
Closeness and Appreciation → Conservation Awareness	0.151	1.453	0.073	Not supported
Closeness and Appreciation → Species Knowledge	0.325	2.342	0.010	Supported
Feeling of Responsibility → Conservation Awareness	0.044	0.654	0.257	Not supported
Conservation Awareness → Species Knowledge	0.528	2.924	0.002	Supported

Proximity and appreciation did not show a significant effect on conservation awareness (β = 0.151; p = 0.073), but had a positive and significant influence on species knowledge (β = 0.325; t = 2.342; p = 0.010). These findings indicate that emotional closeness to a species can directly improve understanding of the species. Sense of responsibility had no significant effect on conservation awareness (β = 0.044; p = 0.257), suggesting that this construct was not yet a determining factor in the models tested. In contrast, conservation awareness has a positive and significant influence on species knowledge (β = 0.528; t = 2.924; p = 0.002), which confirms its role as an important variable in improving understanding of species. These results suggest that not all affective dimensions have a direct effect on species knowledge, and conservation awareness plays a key construct in the structural model tested.

### Indirect effects (Mediation Analysis)

To test the mediating role of conservation awareness, indirect effects were analyzed using bootstrapping as shown in Table 5. The results of the indirect effect analysis using a bootstrapping procedure indicated that conservation awareness plays a selective role as a mediator in the relationship between affective dimensions and species knowledge. Attention and concern show a positive and significant indirect influence on species knowledge through conservation awareness (β = 0.405; t = 2.708; p = 0.003). These findings indicate that the influence of attention and concern for species knowledge operates through an internalization process, resulting in conservation awareness.

**Table 5.** Path coefficient bootstrapping direct effect results.

Indirect Path	β	t-value	p-value	Result
Attention and Concern → Conservation Awareness → Species Knowledge	0.405	2.708	0.003	Supported
Closeness and Appreciation → Conservation Awareness → Species Knowledge	0.080	1.152	0.125	Not supported
Feeling of Responsibility → Conservation Awareness → Species Knowledge	0.023	0.632	0.264	Not supported

n contrast, proximity and appreciation did not show significant indirect influence on species knowledge through conservation awareness (β = 0.080; p = 0.125). This suggests that conservation awareness does not mediate these relationships, so the influence of proximity and appreciation on species knowledge is more direct. In addition, the sense of responsibility did not have a significant indirect influence on species knowledge through conservation awareness (β = 0.023; p = 0.264). These findings reinforce previous results that the sense of responsibility construct did not make a meaningful contribution in the tested model.

## Discussion

### Attention and concern for conservation awareness

The results show that attention and concern have a strong and significant positive influence on conservation awareness, but do not directly affect species knowledge. This highlights the need for targeted educational programs and activities to increase knowledge about the species. ^39^ These findings suggest that attention to a species functions primarily as an affective trigger that enhances awareness, rather than as a means of generating biological knowledge. Previous research has also shown that concern for biodiversity cannot be translated into species-level understanding without cognitive mediation. ^4^ ^,^ ^40^

### Closeness and appreciation for species knowledge

Proximity and appreciation significantly affect species knowledge but not conservation awareness. The results show that proximity to protected forest areas increases species presence and support for conservation, but that support decreases as wildlife-caused damage increases. ^41^ Emotional closeness can facilitate the learning process through increased interest and engagement with species-related information, which supports direct knowledge. Learners who build emotional connectedness through direct interaction with animals and discussions with peers can be encouraged to develop their empathy and curiosity. ^42^ These results are in line with research emphasizing the role of emotional connections in improving learning motivation and ecological understanding. ^43^ Environment-based learning strategies can improve knowledge about species and conservation efforts.

### Feeling of responsibility for conservation awareness

In contrast, a sense of responsibility does not show a significant impact on conservation awareness. If people feel they cannot contribute effectively to conservation efforts, their sense of responsibility may not motivate them to act. ^44^ In addition, awareness of conservation efforts does not necessarily lead to positive attitudes or actions towards conservation. ^45^ These findings support previous evidence that moral responsibility, when not supported by contextual knowledge, tends to be abstract and insufficient to form conservation consciousness. ^17^ Community involvement and government support are needed to raise conservation awareness.

### Conservation awareness for species knowledge

Conservation awareness has a significant positive influence on species knowledge, highlighting its central role in structural models. Studies show that individuals who are more aware of conservation issues directly increase their knowledge of the species. ^11^ Programs that integrate conservation education have been shown to increase knowledge of species significantly. ^46^ Awareness-raising campaigns through educational materials can significantly increase knowledge about the species. In addition, attachment to place and social identity play an important role in driving one’s conservation behavior and knowledge of the species. These factors can improve an individual’s understanding and support for conservation as well as knowledge of species. ^47^ Ultimately leading to more targeted and beneficial decisions and actions for biodiversity.

### Path coefficient bootstrapping direct effect results

Significant influence (β = 0.405; t = 2.708; p = 0.003). These results empirically confirm that attention alone is insufficient to develop species knowledge and that it requires support from conservation awareness. Environmental education, which begins at an early age, can significantly improve knowledge and attitudes towards biodiversity conservation. ^48^ These findings answer research gaps, as the mediating role of conservation awareness has rarely been empirically tested using PLS-SEM. It is necessary to incorporate affective components into educational strategies to improve learning outcomes. ^49^ Mindfulness acts as a bridge between affective sensitivity and cognitive learning outcomes. ^50^ In contrast, conservation awareness does not mediate the effects of proximity, appreciation, or a sense of responsibility towards species knowledge. This is due to a lack of hands-on experience, emotional involvement, and knowledge of the species. ^51^ ^,^ ^52^ Although proximity can directly enhance learning, responsibility requires additional pedagogical support in influencing awareness and knowledge development.

This study has several limitations that need to be considered in interpreting the findings. First, the limitation lies in the use of a cross-sectional survey design, which collects data at a single time point. Hence, the study only describes relationships among variables and cannot ascertain the direction of cause and effect. Second, data collected through questionnaires may be biased, which can affect the research results. Third, the research sample is limited to prospective biology teacher students in the West Nusa Tenggara and East Nusa Tenggara regions, so the generalization of the findings to other geographic contexts or populations should be considered. Fourth, this study focuses on a single local species and therefore does not fully capture the complexity of species literacy within broader biodiversity. In addition, the variables used in the model remain limited to affective dimensions and conservation awareness, and thus do not include other factors that may affect species knowledge, such as field experience and learning strategies.

Future research is recommended to use longitudinal designs, expand the sample size, and integrate additional variables to gain a more comprehensive understanding. In addition, the use of diverse data collection methods, such as direct observation and in-depth interviews, should be considered to minimize potential bias in the questionnaire data. Further research may also involve more than one species to test the consistency of the relationship patterns found and expand the findings in the context of species literacy and conservation education.

## Conclusion

This study shows that the affective dimension and conservation awareness have an important role in shaping species knowledge in prospective biology teacher students. These findings show that attention and concern significantly affect conservation awareness, which in turn is a major determinant of improved species knowledge. Meanwhile, closeness and appreciation have a direct effect on species knowledge without going through conservation awareness, and a sense of responsibility does not show a significant influence.

These findings highlight the role of conservation awareness as a mediator that bridges affective and cognitive aspects. This confirms that affective involvement alone is not enough without being followed by the formation of structured consciousness. This study confirms that the integration between affective and cognitive processes strongly influences the development of species literacy. The implications for the education of prospective biology teachers include strengthening conservation awareness and using local species contexts to improve more meaningful, contextual biodiversity learning.

## Ethical considerations

This research involved human participants in an educational setting. Research Permit was approved by the Faculty of Teacher Training and Education, University of Cordova (approval number: 130/U08.03/PN/2025; date: April 22, 2025). Prior to data collection, all participants received information about the purpose of the study, the learning procedures, the data to be collected, confidentiality, voluntary participation, and the right to withdraw from the study at any time without academic consequences. Written informed consent was obtained from all participants prior to their involvement in the study. No minors were involved in this study.

## Data Availability

The underlying data supporting the findings of this study are publicly available on Zenodo ^53^ at https://doi.org/10.5281/zenodo.21766198. This dataset includes the *Species Literacy Attitude Questionnaire* and the anonymized *Respondent Answers* used to compile the tables in this article. The data are available under the Creative Commons Attribution 4.0 International (CC BY 4.0) license. Not applicable.
