# Supplementary material for: Conservation Awareness as a Key Mediator Linking Affective Factors to Species Knowledge in Pre-Service Biology Teachers 
Source: F1000Res. 2026 Sep 7;15:1488. [Version 1] doi: 10.12688/f1000research.188371.1 (PMC13631440; doi:10.12688/f1000research.188371.1)
Supplement: Peer review history

# Reviewer report: Reviewer response for version 1

**DOI:** 10.5256/f1000research.207894.r520140

**Published:** 2026-10-01

**Authors:** M. Eval Setiawan

**Affiliations:** 1 Insitut Agama Islam Negeri Kerinci, Sungai Penuh, Indonesia

This manuscript addresses a relevant topic in biology education and conservation by examining the relationships among affective factors, conservation awareness, and species knowledge among pre-service biology teachers. The study provides valuable contextual insight through its focus on a local species and its examination of the mediating role of conservation awareness. Overall, the manuscript is well structured, and the findings have potential significance for biology education and conservation. However, several aspects require further clarification and refinement, particularly regarding construct conceptualisation, measurement, interpretation of the results, and consistency of terminology. The following comments are intended to help improve the clarity and rigour of the manuscript.

 Title: The article title is clear, informative, and accurately reflects the primary relationships among the variables examined, particularly the role of conservation awareness as a mediator between affective factors and species knowledge.

 Abstract The abstract presents a comprehensive overview of the study objectives, research design, sample characteristics, analytical methods, and key findings. One suggestion is to refine the wording describing the mediation effects and outcome-related claims so the reported findings remain proportionate to the study design and do not imply conclusions beyond what the data support.

 Background: The introduction offers relevant context regarding the importance of species knowledge, affective factors, and conservation awareness in the education of pre-service biology teachers. The cited literature also provides an adequate basis for the development of the research model. However, a few points could be clarified. In particular, the conceptual relationships among affective factors, conservation awareness, and species knowledge should be made more explicit, especially the theoretical rationale for positioning conservation awareness as a mediator. The authors are also encouraged to clarify the conceptual boundaries between species knowledge, conservation awareness, and the affective constructs to minimise potential overlap among these variables.

 Methods: The Methods provide an adequate description of the research design, participants, instruments, and PLS-SEM analysis. However, several technical details require further clarification, particularly the rationale for sample selection, the characteristics of the instruments, and the justification for the operationalisation of each construct. Particular attention should be given to the measurement of species knowledge. The authors should clarify and justify the use of a Likert-scale instrument for

 this construct, as such an approach appears to capture perceived species knowledge rather than directly assessing factual or objective species knowledge.

 Result: The results are presented systematically, allowing readers to follow the relationships among the variables within the proposed model. The key findings concerning the relationships between affective factors, conservation awareness, and species knowledge are reported clearly. Readability could be improved by placing greater emphasis on the main patterns of the findings rather than reiterating statistical values that are already presented in the tables. The terminology used to describe direct effects, indirect effects, and non-significant pathways should also remain consistent throughout this section.

 Discussion: The discussion appropriately relates the findings to the existing literature and offers interpretations of the observed relationships. This section could be strengthened by moving beyond simply stating whether the findings are consistent with or differ from previous studies and by providing a clearer explanation of the mechanisms that may underlie these relationships. The finding that attention and care are related to species knowledge through conservation awareness, whereas closeness and appreciation show a direct relationship with species knowledge, is particularly interesting and warrants further discussion. The non-significant finding for sense of responsibility also requires a more cautious interpretation, avoiding any implication that this construct is unimportant in the broader context of conservation.

 Conclusions: The conclusion summarises the key findings in a manner that is relevant to and consistent with the study objectives. However, some statements should be framed more cautiously in light of the observational design. The authors are advised to limit their conclusions to the empirical relationships identified in the study and to avoid wording that may imply causality. The implications for the education of pre-service biology teachers may be retained, but they should be presented as potential implications that require further empirical validation.

 General Reviewer Statement: Overall, this article addresses a relevant topic in biology education and conservation and is based on a clearly structured research design. The proposed model linking affective factors, conservation awareness, and species knowledge offers an interesting perspective, particularly in the context of pre-service biology teachers. The analytical approach is applied systematically, and the key findings are presented clearly. Nevertheless, several minor clarifications are required, particularly regarding the precision of construct definitions, consistency of terminology, the interpretation of the measurement of species knowledge, and caution in discussing the implications of the findings. These revisions do not require changes to the core research design or the main analytical approach. Based on these considerations, I recommend minor revision before the manuscript is considered further for publication.

Is the work clearly and accurately presented and does it cite the current literature?

Yes

If applicable, is the statistical analysis and its interpretation appropriate?

Yes

Are all the source data underlying the results available to ensure full reproducibility?

Yes

Is the study design appropriate and is the work technically sound?

Yes

Are the conclusions drawn adequately supported by the results?

Yes

Are sufficient details of methods and analysis provided to allow replication by others?

Yes

Reviewer Expertise:

Biology Education, STEM in Biology,  Biodiversity & Conservation Literacy, and Environmental Education.

I confirm that I have read this submission and believe that I have an appropriate level of expertise to confirm that it is of an acceptable scientific standard.

# Reviewer report: Reviewer response for version 1

**DOI:** 10.5256/f1000research.207894.r520143

**Published:** 2026-09-25

**Authors:** Musa Dikmenli

**Affiliations:** 1 Necmettin Erbakan University, Konya, Turkey

This study aims to integrate models by examining affective dimensions, conservation awareness, and species knowledge within a single framework using Partial Least Squares–Structural Equation Modeling (PLS-SEM), and to test the mediating role of conservation awareness in shaping the species literacy of prospective biology teacher students through the species Lichmera indistincta limbata.  This study highlights that conservation awareness is a key mechanism bridging the affective and cognitive dimensions of species literacy and is important to integrate into the education of prospective biology teachers in the context of local species. It is considered more appropriate to limit and reduce the number of keywords. Why was the *Lichmera indistincta limbata* subspecies selected? What was the primary factor in the selection of this subspecies? A detailed explanation is required. Is it possible to assess genre literacy based on a single genre, even if this is noted as a limitation? 

 Which method was used to determine the sample group? The development of the survey questions used should be explained in greater detail. Were expert opinions sought during the development phase? Was a pilot study conducted? For model fit indices, the acceptable value for NFI is .90 or higher. The value of .83 obtained in this model is considered insufficient. Modifications proposed for the model may be considered. The deleted indicators and reasons for deletion should also be reported.

 While the small number of participants is a limitation of the study itself, it does have an impact on the generalizability of

 the study data. The discussion is written quite comprehensively and references current research are made. If the authors make the necessary corrections, this work is suitable for scientific publication.

Is the work clearly and accurately presented and does it cite the current literature?

Yes

If applicable, is the statistical analysis and its interpretation appropriate?

Yes

Are all the source data underlying the results available to ensure full reproducibility?

Yes

Is the study design appropriate and is the work technically sound?

Yes

Are the conclusions drawn adequately supported by the results?

Yes

Are sufficient details of methods and analysis provided to allow replication by others?

Yes

Reviewer Expertise:

Biology education, misconception, analogy

I confirm that I have read this submission and believe that I have an appropriate level of expertise to confirm that it is of an acceptable scientific standard, however I have significant reservations, as outlined above.

# Reviewer report: Reviewer response for version 1

**DOI:** 10.5256/f1000research.207894.r520139

**Published:** 2026-09-12

**Authors:** Nurwidodo Nurwidodo

**Affiliations:** 1 Universitas Muhammadiyah Malang, Malang, Indonesia

This manuscript addresses a relevant topic by examining conservation awareness as a mediator between affective factors and species knowledge among pre-service biology teachers. The use of a local species provides contextual value. The manuscript is generally understandable and the results are potentially useful; however, several methodological explanations, statistical interpretations, and language issues should be corrected. I recommend **minor revision**, with particular attention to the PLS-SEM reporting.

 Abstract

- The abstract clearly presents the background, methods, main findings, and conclusion.
- Please use the past tense consistently when describing the completed study, for example, “This study employed” and “PLS-SEM was used.”
- Specify the sampling technique used to recruit the 137 respondents.
- The Results section should report the principal coefficients, including the significant indirect effect, rather than presenting only narrative findings.
- Please clarify whether “species knowledge” represents objective knowledge or respondents’ self-reported knowledge measured using Likert-scale items.
- Use consistent construct terminology. The manuscript alternates between “attention and care” and “attention and concern.”

Introduction

- The introduction establishes the importance of biodiversity and species literacy, but some ideas are repeated. For example, the statement concerning bird-feeding and monitoring activities appears twice.
- Several sentences are incomplete or grammatically unclear, such as “Due to the complex levels of ecological understanding…” and “Ultimately leading to more targeted….”
- The transition from the general biodiversity crisis to the selected local bird species should be strengthened.
- Please explain more clearly why *Lichmera indistincta limbata* was chosen and why knowledge of this particular species is important for pre-service biology teachers in the study region.
- The conceptual differences among affective factors, conservation awareness, and species knowledge require clearer theoretical explanation.
- The research gap is relevant but should be stated more precisely. The authors should demonstrate what previous mediation studies have not examined.
- Consider presenting explicit hypotheses, such as H1–H9, corresponding to the direct and indirect relationships tested.
- Scientific names, including *Lichmera indistincta limbata*, should be consistently italicized.

Methods

- Change the subsection title “Participants research” to “Participants.”
- Explain the sampling method, inclusion criteria, respondent recruitment procedure, response rate, and period of data collection.
- Provide a clear justification for the sample size. A power analysis or an established PLS-SEM sample-size procedure would strengthen the study.
- Report the number of questionnaire items for every construct and provide representative items or refer clearly to the complete instrument in the repository.
- Clarify how the instrument was adapted, translated, validated, and pilot-tested.
- The manuscript states that all variables, including species knowledge, were measured using a five-point Likert scale. The authors should discuss whether this measures actual species knowledge or perceived knowledge.
- Provide respondent characteristics beyond sex, such as age, semester, university type, and prior conservation or field experience.
- The measurement-model results should not be presented prematurely in the Data Collection subsection. They belong in the Results section.
- Discriminant validity should be evaluated using HTMT in addition to, or instead of relying exclusively on, cross-loadings.
- Because all data were collected from the same respondents through one online questionnaire, possible common-method bias should be assessed and discussed.
- Report the number of bootstrap subsamples, the use of one- or two-tailed testing, the confidence-interval method, and the handling of missing data.
- The ethical statement is adequate, but it should be stated whether the approving institution functioned as an ethics committee or only issued a research permit.

Results

- Table 1 should report each indicator and its outer loading, not only the loading range. The deleted indicators and reasons for deletion should also be reported.
- Please add HTMT values and confidence intervals to demonstrate discriminant validity.
- The criteria reported for d_ULS and d_G in Table 2 are incorrect or insufficient. These values should be compared with bootstrap-based reference values, rather than interpreted simply as being greater than 0.05.
- The chi-square comparison in Table 2 requires correction and is not adequately justified for PLS-SEM.
- The use of the traditional global GoF index in PLS-SEM is controversial and should either be removed or supported with an appropriate methodological justification.
- An NFI value of 0.827 should not automatically be interpreted as strong model fit without qualification.
- The R² values of 0.894 and 0.837 are unusually high for the proposed behavioural model. The authors should examine possible construct overlap, common-method bias, and discriminant-validity problems.
- The manuscript should report additional structural-model indicators, particularly collinearity statistics, effect sizes (f²), and predictive relevance (Q²).
- There is an important inconsistency between the stated significance criterion and the reported p-values. The Methods specify t>1.96t>1.96, indicating two-tailed testing, but several p-values in Tables 4 and 5 appear to be one-tailed. For example, t=1.453t=1.453 is reported as p=0.073p=0.073. Please clarify and recalculate all p-values consistently.
- Replace “p = 0.000” with “p < 0.001.”
- Table 5 is incorrectly titled “direct effect results”; it reports indirect effects.
- Correct “Variable Dependen” to “Dependent Variable.”
- The mediation results should include bootstrap confidence intervals. The authors should also specify whether the significant mediation is indirect-only, complementary, or another mediation type.
- There is a typographical error at the beginning of the mediation paragraph: “n contrast” should be “In contrast.”
- Several sentences and interpretations are duplicated and should be removed.

Discussion

- The discussion follows the tested relationships, which makes it easy to understand.
- However, parts of the discussion merely repeat the numerical results. The authors should provide stronger theoretical explanations for why some affective dimensions were significant while responsibility was not.
- Avoid causal wording such as “affect,” “influence,” or “increase” because the cross-sectional survey design only demonstrates statistical associations.
- The discussion of “closeness and appreciation” should be more directly related to the selected species and the educational experiences of the respondents.
- The subsection title “Path coefficient bootstrapping direct effect results” is unsuitable because the subsection mainly discusses mediation. Consider using “The Mediating Role of Conservation Awareness.”
- The term “mindfulness” is introduced even though the analyzed construct is conservation awareness. These concepts should not be treated as interchangeable without theoretical justification.
- Some cited studies concern tourists, communities, or schoolchildren rather than pre-service teachers. Explain their relevance and avoid overly broad generalizations.
- Expand the practical implications by describing specific activities that teacher-education programs could implement, such as local-species observation, field identification, citizen-science projects, or conservation-based assignments.
- The limitations section is appropriate, although the use of self-reported species knowledge and common-method bias should be explicitly added.

Conclusion

- The conclusion adequately summarizes the primary findings and educational implications.
- Avoid causal statements and replace them with wording such as “was associated with” or “showed a significant relationship with.”
- The statement that affective and cognitive integration “strongly influences” species literacy is broader than the evidence supports.
- Briefly emphasize the study’s principal contribution: the selective mediating role of conservation awareness between attention/concern and species knowledge.
- The conclusion can be shortened to avoid repeating the Results and Discussion sections.

Data availability and reporting

- The Zenodo repository is a positive aspect of the manuscript.
- Please ensure that it includes the complete questionnaire, anonymized raw data, variable codebook, scoring instructions, excluded indicators, and analysis output or project files.
- “Reporting guidelines: Not applicable” should be reconsidered. The authors may provide an appropriate checklist for cross-sectional observational research if required by the journal.

References

- The reference list is sufficiently extensive and generally relevant, but formatting and bibliographic completeness should be carefully checked.
- Several references lack essential details such as page ranges, issue numbers, publishers, or DOI information.
- Reference 1 appears only indirectly related to the manuscript’s central topic and should be replaced with a stronger source on biodiversity loss or ecosystem resilience.
- References used to justify PLS-SEM procedures should prioritize authoritative methodological sources.
- Some methodological references are drawn from unrelated applied fields. More direct PLS-SEM sources would provide stronger justification.
- Check consistency in journal-title abbreviations, capitalization, author initials, publication dates, and use of “et al.”
- Verify that every reference supports the specific statement to which it is attached.
- Correct the spelling of “Supplementary” in Reference 53, currently written as “Suplementary.”
- Ensure that the Zenodo record contains complete metadata and matches the final article title.

Recommendation

**Minor revision.** The topic is relevant and the manuscript has publication potential, but the authors should correct the PLS-SEM significance testing, improve measurement-model reporting, clarify the nature of the species-knowledge construct, and undertake careful English-language editing.

Is the work clearly and accurately presented and does it cite the current literature?

Yes

If applicable, is the statistical analysis and its interpretation appropriate?

Yes

Are all the source data underlying the results available to ensure full reproducibility?

Yes

Is the study design appropriate and is the work technically sound?

Yes

Are the conclusions drawn adequately supported by the results?

Yes

Are sufficient details of methods and analysis provided to allow replication by others?

Yes

Reviewer Expertise:

Ecology and Environmental education

I confirm that I have read this submission and believe that I have an appropriate level of expertise to confirm that it is of an acceptable scientific standard.
